# Trends in Women’s Leadership of Oncology Clinical Trials

**DOI:** 10.3389/fonc.2022.885275

**Published:** 2022-06-08

**Authors:** Ithai Waldhorn, Ayelet Dekel, Anna Morozov, Elisa Sardas Alon, Danielle Stave, Noam Ben Tsrooya, Shir Schlosser, Gal Markel, David Bomze, Tomer Meirson

**Affiliations:** ^1^ Division of Oncology, Rambam Health Care Campus, Haifa, Israel; ^2^ Midaat - For Informed Health, Mevaseret Zion, Israel; ^3^ Department of Data Science, Eyeviation, Ramat Gan, Israel; ^4^ The Israel Society for Gender and Sex Conscious Medicine, Tel Aviv, Israel; ^5^ Department of Pediatrics, Dana Dwek Children’s Hospital, Tel Aviv Sourasky Medical Center, Tel Aviv, Israel; ^6^ Occupational Medicine Department, Clalit Health Services, Netanya, Israel; ^7^ Sackler Faculty of Medicine, Tel Aviv University, Tel Aviv, Israel; ^8^ Davidoff Cancer Center, Rabin Medical Center-Beilinson Hospital, Petah Tikva, Israel

**Keywords:** women representation, women’s leadership, gender gap, oncology clinical trials, principal investigators

## Abstract

It has been widely reported that women are underrepresented in leadership positions within academic medicine. This study aimed to assess trends in women representation as principal investigators (PIs) in oncology clinical trials and to characterize trends in women’s leadership in such trials conducted between 1999 and 2019. The gender of 39,240 PIs leading clinical trials was determined using the gender prediction software Genderize.io. In total, 11,516 (27.7%) women served as PIs. Over the past 20 years, an annual increase of 0.65% in women PIs was observed. Analysis by geographic distribution revealed higher women representation among PIs in North America and Europe compared to Asia. Industry-funded trials were associated with lower women PI representation than academic-funded trials (31.4% vs. 18.8%, p<0.001). Also, women PIs were found to be underrepresented in late-phase as compared to early-phase studies (27.9%, 25.7%, 21.6%, and 22.4% in phase I, II, III, and IV, respectively; Cochran-Armitage test for trend, p<0.001). Furthermore, an association was found between the PI’s gender and enrolment of female subjects (50% vs. 43% female participants led by women vs men PIs, respectively, p<0.001). Taken together, while the gender gap in women’s leadership in oncology trials has been steadily closing, prominent inequalities remain in non-Western countries, advanced study phases, industry-funded trials and appear to be linked to a gender gap in patient accrual. These observations can serve for the development of strategies to increase women’s representation and to monitor progress toward gender equality in PIs of cancer clinical trials.

## Introduction

Over the past few decades, women have made substantial gains in participation in the medical profession. As of 2020, women represented 34% of practicing physicians and 50.5% of medical students within the United States (AAMC 2019 Physician Specialty Data Report). However, the underrepresentation of women remains prevalent in science and medicine. A growing body of literature has shown an achievement gap between men and women faculty in research practices, career advancement, leadership opportunities, financial compensation, and scientific recognition (1–6).

In oncology, women are estimated to represent 36% of the workforce (7) but account for only one-fifth of full professors and one-third of department leaders (8). Despite positive trends, the percentage of women among authors in major oncology journals remains low (20-30%), lagging behind the proportions serving as oncology faculty (9, 10). In addition, women represented ~40% of invited speakers in oncology international congresses and one-third of board members of oncology societies (11, 12).

Clinical trials are the backbone of evidence-based medicine and promote informed clinical decision-making. They require infrastructure, advanced research training, and massive funding, and take years from planning to completion. Serving as a principal investigator (PI) in a clinical trial confers recognition among peers and at international oncology meetings, and can result in academic promotion. Therefore, being a PI is a major milestone in an oncologist’s career.

In this study, we aimed to assess women’s representation as PIs in oncology clinical trials, characterize trends, and determine factors associated with women leadership.

## Materials and Methods

### Study Selection

**Figure 1:**
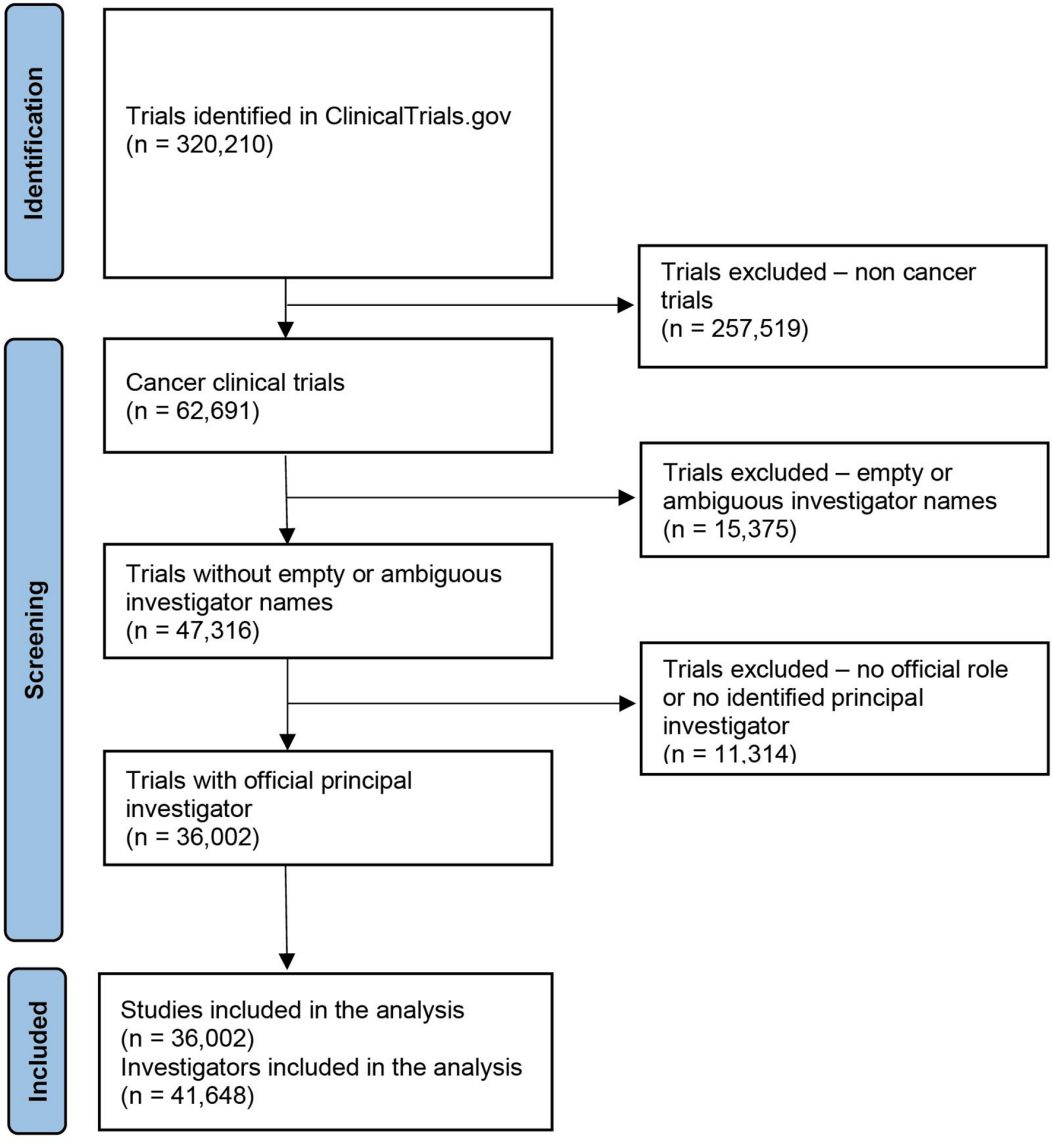
Flow chart of trial screening and eligibility.

Study record data were downloaded from ClinicalTrials.gov in extensible markup language (XML) format on October 24, 2019. This search yielded 320,210 trials conducted between January 1999 and October 2019 (**Figure 1**). Trial data, including ClinicalTrials.gov identifier, year of submission, investigator names, investigator role, study phase, study type, sponsorship, affiliation, Medical Subject Headings (MeSH) term, and the number of male and female participants in the study, were abstracted. The analysis was restricted to oncology trials by including studies matching the MeSH term “Neoplasms”. Studies with empty or ambiguous PI names were excluded, and only investigators with an official role assigned as “Principal Investigator” were included in the analyses.

The study and reporting followed Preferred Reporting Items for Systematic Reviews and Meta-Analyses (PRISMA) guidelines (13).

### Determination of Principal Investigator Gender

First names were subjected to basic processing to remove extra spaces, ambiguous characters, and prefixes such as doctor (e.g., Dr.) or professor (e.g., Prof.). The PI gender, treated for the purpose of this analysis as binary (i.e., woman or man), was predicted using the validated software Genderize.io (https://genderize.io). For each name, the software returns a predicted gender and a probability. The standard threshold of 60% was used to assign the gender, as has been implemented in previous works (14–16). Names predicted with a lower probability, for which prediction failed, or which were ambiguous were marked as not applicable.

### Geocoding

Google Maps API through the R package mapsapi version 0.5.0 was used to locate the country of the PI. Since a given trial may be led by more than one investigator and the documented country in the study records is not necessarily the country of the PI, the affiliation of the investigators was used for geocoding (e.g., Department of Family Medicine, University of Michigan). Countries with fewer than 30 studies were excluded from the analysis. Countries were classified as low/lower-middle, upper-middle and high-income based on their World Bank Classification.

### Statistical Analysis

All analyses were conducted in R version 4.0.3 (R Project for Statistical Computing). Odds ratios (OR) were estimated by logistic regression using the R package glm. The Cochran–Armitage trend test was used to estimate the association between representation of women PIs over time and study phases using the R package CATT. The association between the genders of the PI and participants was evaluated using the Wilcoxon rank-sum test. Two-sided p values <0.05 were considered statistically significant.

### Ethical Approval

Institutional review board approval was waived because no human data were included, and publicly available information was used.

## Results

**Figure 2:**
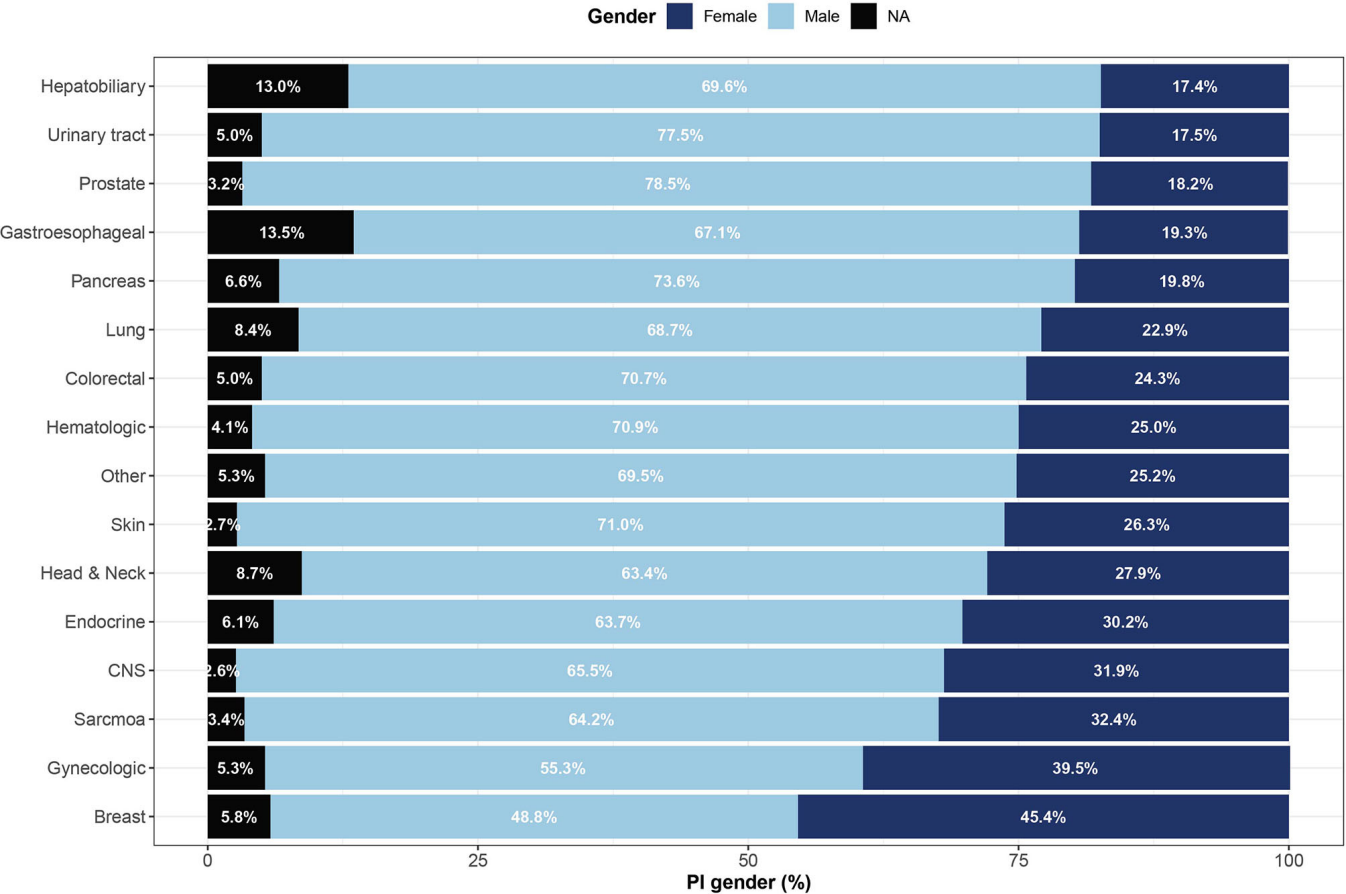
Representation of women among lead investigators of oncology clinical trials by cancer site. Shown are gender proportions of principal investigators leading studies of 16 cancer sites. Names with low gender prediction scores or names for which gender could not be determined are marked as not applicable (NA). CNS, central nervous system.

The online system ClinicalTrials.gov is a web-based resource that provides access to summary information about ongoing and completed clinical studies. Out of 320,210 trials registered in ClinicalTrials.gov between 1999 and 2019, we identified 36,002 unique cancer clinical trials led by 41,648 PIs (**Figure 1**). The gender of 39,240 (94.2%) investigators could be determined. In total, 11,516 (27.7%) women served as PIs in cancer clinical trials, compared to 27,724 (66.6%) men. Categorizing by cancer disease site found low women leadership rates for hepatobiliary (17.4%), urinary tract (17.5%), prostate (18.2%) and gastroesophageal (19.3%) cancer trials and higher rates for breast (45.4%), gynecologic (39.5%), sarcoma (32.4%), central nervous system (31.9%) and endocrine (30.2%) cancer clinical trials (**Figure 2**).

**Figure 3:**
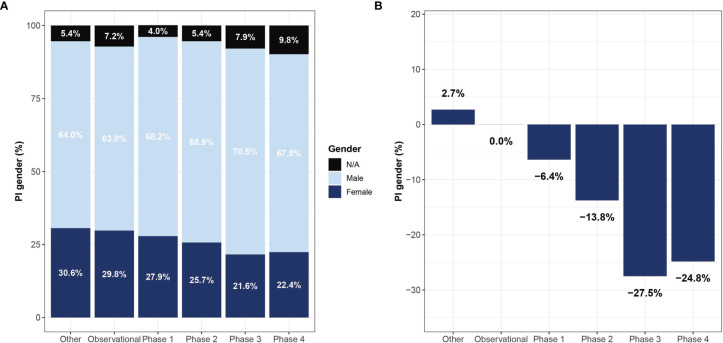
Representation of women lead investigators in oncology clinical trials by study type. Shown are gender proportions of principal investigators leading trials of different study phases **(A)** and the reduction in proportions relative to observational studies **(B)**. Names with low gender prediction score or names who which gender could not be determined are marked as not applicable (NA).

**Figure 4:**
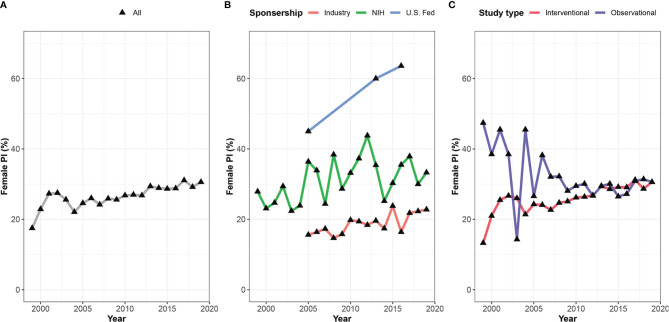
Gender gap among lead investigators in oncology clinical trials over time. Shown are proportions of women leading oncology trials between 1999 and 2019 for **(A)** all included studies, **(B)** studies stratified by sponsorship, and **(C)** type of study.

A significant association was found between the clinical trial phase and proportions of women PIs, where late phases were led by fewer women compared to early phases – 27.9%, 25.7%, 21.6%, 22.4% in phase I, II, III, and IV, respectively (Cochran-Armitage test for trend, p<0.001, **Figure 3**). Observational trials had more women PIs than interventional trials (29.8% vs. 27.2%; OR 1.27, 95% CI, 1.27 – 1.35; P<0.001). This disparity was most apparent in phase III clinical trials (Phase III vs observational studies, OR 1.54, 95% CI, 1.40 – 1.70; p<0.001). In addition, a significant relationship between study sponsorship and the gender of the PI was observed, where fewer clinical trials led by women were funded by the industry (18.8%) as compared with those funded by the NIH (31.4%; OR 0.53, 95% CI, 0.48 – 0.60; p<0.001) or US federal agencies (34.8%; OR 0.46, 95% CI, 0.32 – 0.66; p<0.001) (**Figure 4**). Over time, there was a substantial increase in women’s leadership of clinical trials from 17.5% in 1999 to 30.6% in 2019 (5-year interval: 17.5%, 22.1%, 25.6%, 28.9%, 30.6%), representing an average annual increase of 0.65% (**Figure 4**). A Cochran-Armitage trend test found this growth of women PI’s proportion to represent a steady and significant increase over time (p=0.001). Women’s leadership increased in both industry and academic-funded trials (**Figure 4B**).

**Figure 5:**
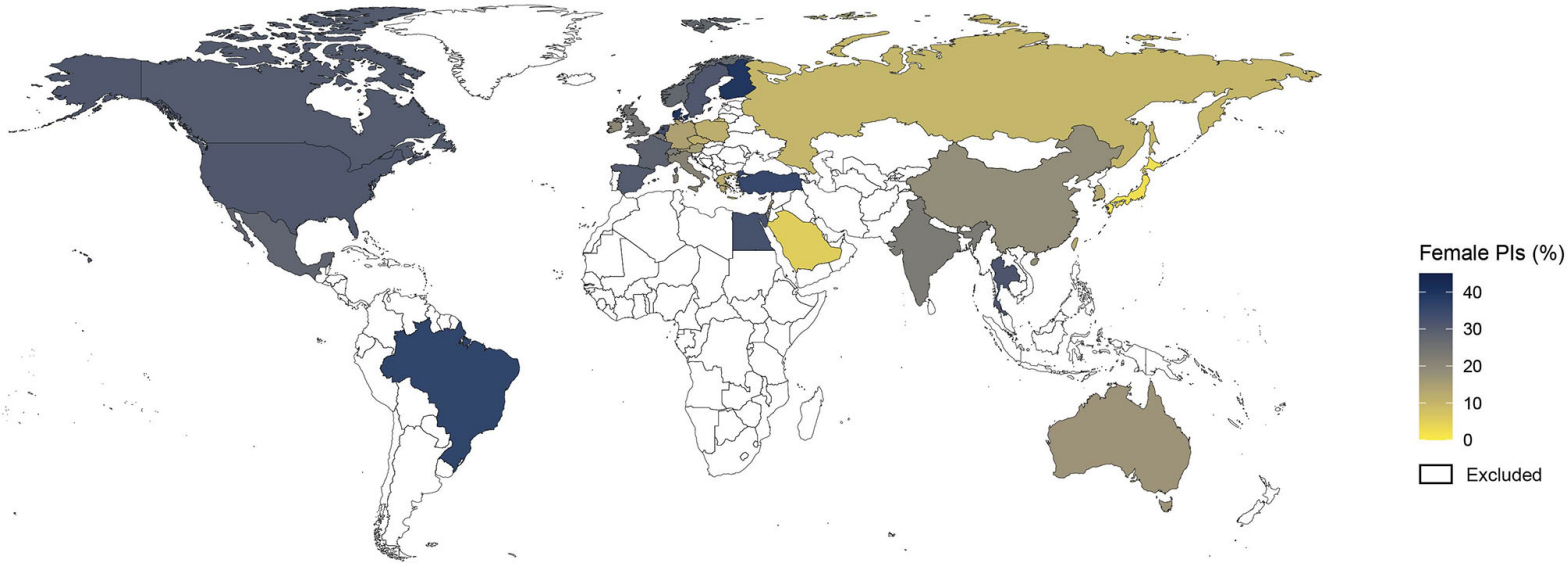
Map of the gender gap among lead investigators of cancer clinical trials. Shown is the distribution of proportions of women principal investigators (PIs) by country. Countries with less than 30 clinical trials are colored in white and were excluded from the analysis.

Analysis by geographic distribution revealed higher women representation among PIs in North America (30.7%) and Europe (23.8%) compared to Asia (15.5%), although the rates of women PIs varied across European countries (**Figure 5** and **Supplementary Table 1**). For example, Denmark (39.3%), Sweden (31.1%) and France (28.6%) had higher women representation than Germany (14.2%), Italy (21.3%) and Austria (15.5%). A comparison according to the level of resources showed higher representation of women PIs among high-income countries compared with middle-income countries (28.6% vs 20.5%).

**Figure 6:**
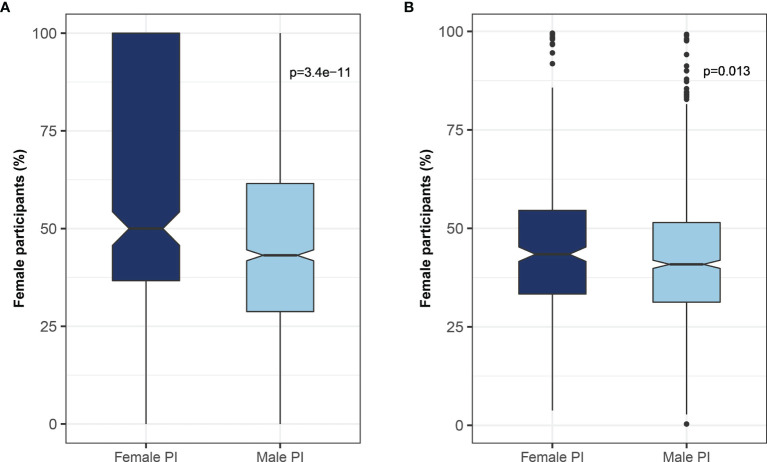
Relationship between the gender of the investigator and trial participants. The association between the gender of the lead investigator and the proportion of female participants enrolled in the trial for **(A)** all studies, and **(B)** studies excluding gender-specific malignancies (e.g., uterine, ovary, and prostate).

It was previously shown that women are underrepresented as study participants in clinical trials (17). We found that men leading clinical trials were less likely to recruit women participants, whereas women leading clinical trials tended to recruit more women participants (50% vs. 43%, female participants led by women vs men PIs p<0.001) (**Figure 6**). This observation remained statistically significant even after excluding gender-specific diseases such as breast, prostate, and gynecologic malignancies (44% vs. 41% p-value 0.013) (**Figure 6**). Of note, only 1,749 (4.9%) studies reported the number of women and men participants in the trial.

## Discussion

This study of gender representation in cancer clinical trials found that while men lead the majority of clinical trials, women representation among PIs is growing. Women’s leadership of clinical trials is more prevalent in Western countries, early-phase trials, and nationally sponsored studies. In addition, clinical trials led by women PIs have a greater representation of female study participants.

It was previously shown that women are a minority among first authors in cancer-related publications, oncology faculty members, subjects of phase III randomized clinical trials, invited speakers, and board members of oncology societies (7, 11, 12, 18). To the best of our knowledge, this is the first study to comprehensively evaluate women’s representation among registered cancer clinical trials and trends in women leadership. Interestingly, similar findings were found in other fields as well (19, 20) and may represent a more general phenomenon.

Multiple factors may underlie women’s underrepresentation as PIs. First, women remain a minority in many medical fields. For example, the urologic oncology workforce is primarily comprised of men (21), and women radiation oncologists in genitourinary cancer are a minority (22). This gender gap might affect the observed lower representation of women PIs. Moreover, previous publications have demonstrated marked disparity in the proportion of women in high academic positions (7), board members of oncology societies (11) and as authors in major oncology journals (23). Women comprise 31% of department chairs in medical oncology, 11.7% in radiation oncology and 3.8% in surgical oncology (7). In addition, major oncology societies (ESMO, ASCO) have low percentages of women occupying board position (14-25%). As the oncology field progresses towards gender equality in career development (12), better representation for women as PIs is anticipated.

It will be of great interest to follow the gender gap in clinical trial leadership as the proportion of women leaders increases.

The proportion of women PIs in industry-funded trials was significantly lower than in governmental-funded trials. An earlier study found that 75% of the physicians who had financial relationships with biomedical companies were men (24). Similar results were reported specifically for radiation oncologists (25).

The observed gender disparities in industry-funded trials are in line with gender discrimination and inequality in the general and health workforce (26). We also examined the relationship between the investigator’s gender and women enrollment. Our results demonstrated that clinical trials led by women had higher female subject enrollment. This observation is in accordance with previous studies (27, 28), and supports the notion that reducing the gender gap in women leadership may assist closing the gender gap in recruitment.

The strengths of this study include the longitudinal and comprehensive evaluation of gender representation of PIs in cancer clinical trials. Evaluation of factors associated with gender representation including time trends, study phase, oncology field, sponsorship, and gender of study participants provides a broader prospective on the PIs gender gap. Several limitations of this study warrant mention. First, gender was assumed to be binary (male and female) as in previous studies. The study used validated methods to determine PI's gender, but misclassifications may have occurred. Manual validation of the prediction performance in several countries was performed by random sampling of the predicted genders of names. In addition, this analysis did not account for the proportion of women oncologists in each country and their academic rank. Further, only a small number of oncology trials contained information about the number of participants for each gender. Finally, the observational nature of the study precluded inference of causal relationships.

In conclusion, the present work shed light on trends in women’s leadership in cancer clinical trials over the past two decades. While women comprise a growing proportion of PIs in cancer clinical trials, they remain in the minority. Our findings show significant differences between oncology fields, geographical regions, study phases, and funding agencies. The presented results are important for developing practices and strategies to promote gender equality in the leadership of clinical trials in oncology.

## Data Availability Statement

The original contributions presented in the study are included in the article/**Supplementary Material**. Further inquiries can be directed to the corresponding author.

## Author Contributions

Concept and design: IW, DB, TM. Acquisition, analysis, or interpretation of data: DB, IW, AD, AM, ES, DS, NT, SS, GM, TM. Drafting of the manuscript: IW, TM. Statistical analysis: DB, TM. Supervision: TM. All authors contributed to the article and approved the submitted version.

## Conflict of Interest

AM was employed by Eyeviation, TM reports receiving personal fees from TyrNovo, outside the submitted work. GM reports receiving personal fees from MSD and Roche; grants and personal fees from BMS and Novartis; personal fees and stock options from 4C Biomed; and stock options from Nucleai, Biond Biologics, and Ella Therapeutics, outside the submitted work.

The remaining authors declare that the research was conducted in the absence of any commercial or financial relationships that could be construed as a potential conflict of interest.

## Publisher’s Note

All claims expressed in this article are solely those of the authors and do not necessarily represent those of their affiliated organizations, or those of the publisher, the editors and the reviewers. Any product that may be evaluated in this article, or claim that may be made by its manufacturer, is not guaranteed or endorsed by the publisher.
